# Adenocarcinoma Arising within a Colonic Diverticulum in a Patient with Recurrent Diverticulitis

**DOI:** 10.4137/cmo.s693

**Published:** 2008-08-24

**Authors:** A. van Beurden, C.I.M. Baeten, C.P.E. Lange, H. Doornewaard, L.N.L. Tseng

**Affiliations:** 1 Department of Surgery, Groene Hart Ziekenhuis, Gouda, The Netherlands; 2 Department of Pathology, Groene Hart Ziekenhuis, Gouda, The Netherlands

**Keywords:** adenocarcinoma, diverticulum, colon

## Abstract

In 2006, while admitted in our hospital for surgical treatment of recurrent diverticulitis, a 54-year-old man was found to have an adenocarcinoma arising within a colonic diverticulum. Computed tomography, during this episode of diverticulitis, showed a thickened wall of the sigmoid and inflammatory induration of the pericolonic fat. Colonoscopy could be performed up to no more then 25 cm from the anus due to mucosal edema. A sigmoid resection was performed. Histopathological examination of the resected specimen showed an inflamed diverticulum with a submucosal adenocarcinoma of the intestinal type within its wall. The surrounding flat colonic mucosa was not involved by the cancerous process. Due to lymph node involvement the patient received adjuvant chemotherapy and remained disease free during follow up.

## Introduction

Diverticulitis is common, though most patients remain asymptomatic. Only one-third of the patients who experience an attack of diverticulitis will have chronic or recurrent symptoms.^[1]^ Chronic and recurrent diverticulitis are often treated by resection of the affected bowel part. Malignant tumor development within a diverticulum is distinctly rare and difficult to diagnose. We report a case of adenocarcinoma arising in a diverticulum in a patient with recurrent diverticulitis.

## Case Report

A 54-year-old man presented with recurrent diverticulitis since 1993. From January to August 2006 he suffered from chronic diverticulitis. Computed tomography (CT), during an acute episode in 2006, showed a severely thickened wall of the sigmoid and inflammatory induration of the pericolonic fat tissue, consistent with a diagnosis of diverticulitis. Colonoscopy could only be performed up to 25 cm from the anus due to mucosal edema. A sigmoid resection with side-to-end anastomosis was electively performed. Peroperatively, an obvious diverticulitis was noticed with severe induration of the surrounding tissue. Because the inflamed tissue was fixed to the retroperitoneum, peri- and paracolic lymph nodes were also dissected.

**Figure 1:**
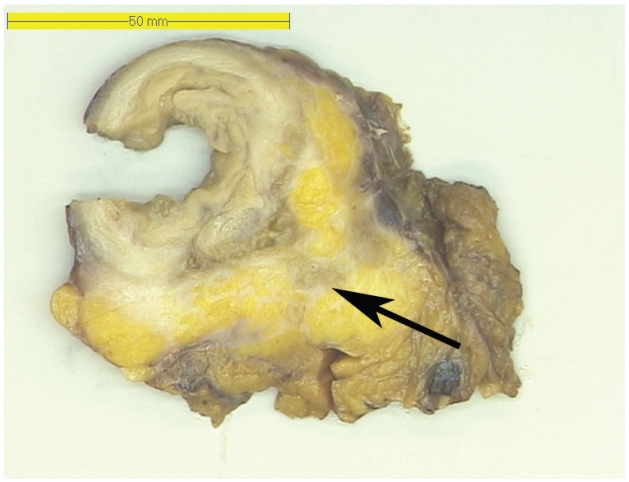
This picture shows a macroscopic view of a colon diverticulum. The fatty subserosal tissue contains some fibrous strands, which microscopically showed adenocarcinoma (arrow).

**Figure 2:**
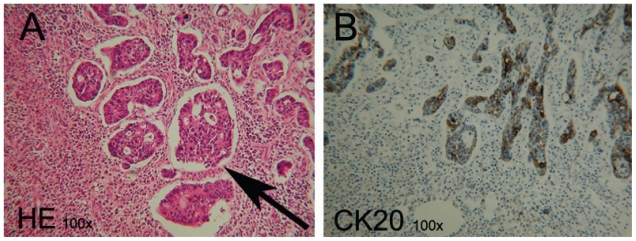
Microscopic examination of the resected specimen: a submucosal adenocarcinoma of the intestinal type, moderately differentiated, within the wall of a diverticulum **A**) In this section (HE-stained) the tumor cell cloths are marked by an arrow. **B**) A serial section shows Cytokeratin 20 positive staining (marker for adenocarcinoma).

Macroscopic examination of the resected specimen showed a 16 cm long specimen, with a diameter of 5 cm and mesenterial fat tissue of 7 cm width. At the outer surface several hemorrhagic areas were observed. On transverse slides multiple diverticula were seen and a number of fibrous strands within the fatty subserosal tissue (Fig. 1). The mucosa did not show any pathological lesions. Microscopic examination of the resected specimen revealed an inflamed diverticulum with a moderately differentiated submucosal adenocarcinoma of the intestinal type, in its wall (Fig. 2). This adenocarcinoma, approximately 2 cm in diameter, infiltrated the subserosa and showed moderate cellular atypia. The surrounding flat colonic mucosa was not involved by the cancerous process. Furthermore 4 of the 12 dissected lymph nodes showed tumor metastases. Immunohistochemical examination excluded the possibility of a metastasis from a primary non-colorectal malignancy (CK7−, CK20+, CEA+, PSA−, and CD56−). An additional chest radiograph and ultrasound of the liver showed no metastases. The postoperative carcinoembryonic antigen (CEA) level was 5.2 μg/l (normal 0–5.2). Due to lymph node involvement, the patient received adjuvant chemotherapy. Oxaliplatin 130 mg/m^2^ was administered eight times in a 3-week cycle; Capecitabin 1000 mg/m^2^ was administered twice a day at day 1 and 14 of each cycle. During one year follow-up there were no signs of metastases or recurrent disease.

## Discussion

Although colonic diverticulitis and cancer are common diseases in Western countries, cancer arising within a diverticulum is rare. In the present report, histopathological examination of the colonic specimen showed a carcinoma within a diverticulum without involvement of the colonic mucosa. Only eight such cases have previously been reported in the literature.^[2]^

Because the occurrence of a carcinoma within a diverticulum is an uncommon phenomenon, this diagnosis can be easily missed. Nowadays CT scan is the technique of choice for assessment of diverticulitis and colon cancer. However, difficulties in distinguishing between these two diseases are well recognized.^[3–5]^ A thickened wall of the sigmoid is a characteristic sign for as well diverticulitis as colon carcinoma.^[6]^ A previous study to distinguish diverticulitis from carcinoma demonstrated that mesenteric inflammation (fluid at the root of the mesentery and vascular engorgement) had a positive predictive value for diverticulitis.^[3]^ Furthermore, the presence of pericolic lymph nodes may suggest a diagnosis of colon cancer rather than diverticulitis.^[4]^ In the current report, we retrospectively were not able to detect any of these specific CT signs.

Although colonoscopy is the standard diagnostic method to detect colon carcinoma, it is not the option of choice in acute diverticulitis. In our patient colonoscopy was performed up to 25 cm from the anus without detecting any signs of carcinoma. And, as the 16 cm long resection included the proximal rectum, we can assume that the colonoscope passed the affected area. Since the surrounding flat mucosa was not involved in the cancerous process this carcinoma was not visible during colonoscopy. Fortunately, we performed a resection with oncological margin due to severe inflammation.

This report demonstrates that a malignancy in a diverticulum can easily be missed during diagnostic work-up for diverticulitis. And, since specific markers to distinguish between diverticulitis and malignant disease are lacking, it remains important to consider the possibility of a cancerous process masquerading as diverticular bowel thickening or phlegmon, especially when surgical intervention is not warranted.
